# Trends in misoprostol use and abortion complications: A cross-sectional study from nine referral hospitals in Nigeria

**DOI:** 10.1371/journal.pone.0209415

**Published:** 2018-12-31

**Authors:** Folasade Adenike Bello, Bukola Fawole, Babawale Oluborode, Ibraheem Awowole, Theresa Irinyenikan, David Awonuga, Olabisi Loto, Adetokunbo Fabamwo, Philip Guest, Bela Ganatra

**Affiliations:** 1 Department of Obstetrics and Gynaecology, University of Ibadan, Ibadan, Nigeria; 2 Department of Obstetrics and Gynaecology, Obafemi Awolowo University Teaching Hospital Complex, Ilesha, Nigeria; 3 Department of Obstetrics and Gynaecology, Obafemi Awolowo University, Ile-Ife, Nigeria; 4 Department of Obstetrics and Gynaecology, State Specialist Hospital, Akure, Nigeria; 5 Department of Obstetrics and Gynaecology, Federal Medical Centre, Abeokuta, Nigeria; 6 Department of Obstetrics and Gynaecology, Lagos State University College of Medicine, Ikeja, Lagos, Nigeria; 7 Institute for Population and Social Research, Salaya, Bangkok, Thailand; 8 UNDP-UNFPA-UNICEF-WHO-World Bank Special Programme of Research, Development and Research Training in Human Reproduction (HRP), World Health Organization, Geneva, Switzerland; Greenebaum Cancer Center, Institute of Human Virology, University of Maryland School of Medicine, UNITED STATES

## Abstract

**Objective:**

The study aimed to assess the use of misoprostol and complications associated with abortions in referral hospitals in Nigeria, a country with restrictive abortion laws.

**Methods:**

A cross-sectional study at nine referral hospitals in South-west Nigeria. Nine years’ data were retrieved from medical records, including 699 induced abortions. Independent variable was the method of abortion; dependent variables were complications, need for treatment and mortality. Statistical significance was tested with Chi-square, Fishers’ exact and chi-square for trend tests (p<0.05).

**Results:**

There were 699 induced abortions amongst 2,463 abortions found in records. Nearly 70% were surgical abortions, but misoprostol use significantly increased over the study period in a linear trend (Χ^2^ trend: 30.96, P <0.001). Patients who used misoprostol were significantly less likely to have infectious morbidity, genital tract injuries or medical complications. There was no difference in incomplete abortion in the groups. Patients were more likely to have in-patient care with surgical abortions (p<0.001), to need prolonged antibiotic regimens (p = 0.003), need further surgeries or additional specialist care (p = 0.009).

**Conclusion:**

Misoprostol abortion has significantly increased over time, and was associated with less morbidity and need for further treatment, in this study. It appears to be the safer option.

## Introduction

A woman’s sexual and reproductive health rights include the ability to have a satisfying and safe sex life while retaining the liberty to prevent pregnancy if it is undesirable [1]. Yet, in Nigeria, the unmet contraceptive need for married women and sexually active unmarried women are 16.1% and 21.8%, respectively [2]. Contraception is not always available or acceptable, and even when used, there are occasional failures associated with its use, resulting in unplanned pregnancies, for which women at times seek abortions. Even when contraception is available, it is apparent that a lot of advocacy is still required: some women consider contraception to be unsafe, avoid using it, and paradoxically consider seeking an abortion for an unwanted pregnancy to be safer than preventing it with a family planning method [3,4]. Women in countries like Nigeria, which have restrictive abortion laws, end up seeking illegal abortions, which unfortunately are often unsafe, because those medically qualified to do them usually do not [5].

Despite the clandestine nature of these abortions, the prevalence in Nigeria remains rather high. In 2012, 56% of unwanted pregnancies ended up in abortions; estimated to be about 1,250,000 abortions [6]. Figures range widely, depending on the population studied: 3.25% of university undergraduates, [7]; 33.5% of women surveyed from the community [8] and 62% of women surveyed at a health care facility [9] had previously had an induced abortion. Seventy-eight per cent of women who were attending an antenatal clinic gave a history of a previous induced abortion [10]. Reported complications rates are expectedly high: post-abortion complications requiring post-abortion care (PAC) occur in 41.4%–72.7% of abortions [9,11,12], yet many do not access this PAC [12]. Two local medical facility-based studies of maternal deaths found that 11.5% and 30.8%, respectively could be attributed to abortion [11,13].

### Rationale

Post-abortion care is expensive; it has been shown to cost six times more than the procedure done for a voluntary termination of pregnancy [14]. Safely-done legal abortions would obviously be a better alternative, but as this is not feasible, inexpensive options for PAC are necessary. A significant part of PAC is directed towards completing an incomplete abortion. Misoprostol, a Prostaglandin E1 analogue which was originally manufactured to treat peptic ulcer disease, can be used to induce or complete abortions because it softens the cervix and causes uterine contractions. The latter also makes it useful in preventing and treating post-partum haemorrhage. It is for these oxytocic effects that it is listed as an essential medicine by World Health Organization (WHO) [15]. Misoprostol is licensed in Nigeria for its off-label use in the management of post-partum hemorrhage in the community, and for PAC at health facilities [16]. It is often available only on prescription (though this restriction is inconsistent), to limit its use as an abortifacient. Anecdotal reports, however, indicate that, it is increasingly being used as an abortifacient in the community, despite this. The study was part of a research in five countries where legal abortion is restricted.

### Objective

This study aimed to determine the types of complications arising from unsafe abortion in Nigeria, the trend in the use of misoprostol for induced abortion, and the relationship between the community use of misoprostol and the complications recorded.

## Materials and methods

This was a cross-sectional study carried out at nine selected secondary and tertiary hospitals which offer post-abortion care in South-West Nigeria. Participating hospitals were Lagos State University Teaching Hospital (LREC/10/06/228); Lagos Island Maternity Hospital (LREC/10/06/228); Sacred Heart Catholic Hospital, Abeokuta (UI/UCH/11/0258); Adeoyo Maternity Teaching Hospital, Ibadan (AD13/479/257); Ring Road State Hospital, Ibadan (AD13/479/257); State Specialist Hospital, Akure (UI/UCH/11/0258); Obafemi Awolowo University Teaching Hospitals Complex, Ile-Ife (ERC/2012/12/01); Obafemi Awolowo University Teaching Hospitals Complex, Ilesa (ERC/2012/12/01) and University College Hospital, Ibadan (UI/UCH/11/0258). Research approval was obtained from the Institutional Research Boards covering all the participating centers (approval reference numbers inserted in parentheses) and from the WHO Ethics Review Committee.

Hospital medical records were the source from which, over a nine-year period from 2006 to 2014, data were retrieved on clinical presentations, methods used in induced abortion, treatment and outcome of management of patients with abortion at each facility, using a 58-item instrument. These records were identified from all possible points of care: emergency room, gynecology clinic, ward and theatre registers, to reduce potential bias on the subjects recruited. There was no pre-specified sample size; all eligible women with abortion complications were included. The independent variable was the method of abortion used. Pelvic instrumentation performed by manual vacuum aspiration or dilatation and curettage was termed as ‘surgical abortions’, as differentiation between them (from the patients’ account) was anticipated to be difficult. Participants mainly identified misoprostol as ‘Cytotec’ or by its polygonal tablet form. Dependent variables were complications of abortions, the need for additional treatment and maternal mortality. Data were analyzed with IBM SPSS Statistics 20 (IBM, Armonk, NY, USA). Statistical significance was set at p<0.05 and tested with chi-square, Fisher’s exact and Χ^2^ for the trend tests.

It was anticipated that some variables might be missing from the data sources, on account of the retrospective design. However, the retrospection was necessary to assess the trends in the previous years, as misoprostol became more available to abortion seekers.

## Results

There were 2,463 cases of abortions during the period of review. Information on the history of interference with the pregnancy was not available in 10 cases, leaving 2,453 cases for consideration. Of these, 1,754 (71.5%) were spontaneous abortions; final analyses were therefore based on the remaining 699 (28.5%) women with induced abortions.

Table 1 shows the methods used in procuring the abortions. Most of the women (484; 69%) had surgical abortions, followed by misoprostol use. Some of the other methods used included: concoctions made up of lime, gin, aspirin and other non-steroidal anti-inflammatory drugs; oxytocics and other injections, oral or vaginal herbal preparations, blunt abdominal trauma, and mixtures of battery water (which may contain some sulphuric acid), detergents or household bleach. No one had used mifepristone.

**Table 1:** Methods used to procure abortion.

Method	Frequency (% of induced abortions)^a^
Misoprostol	87 (12.4)
Surgical abortion	484 (69.2)
Other tablets	130 (18.6)
All other methods	130 (18.6)

^**a**^ Totals exceed 100%, as some participants used more than one method

Table 2 shows that over the study period, the proportion of women using misoprostol for induced abortions rose relative to the other methods (Χ^2^ trend: 30.96, P <0.001). This observed increase in misoprostol abortion, during the study period was a linear increase (Χ^2^_GOF_ 10.19; p = 0.178).

**Table 2:** Trends in methods of induced abortions.

Year	Frequency	
	Induced abortion (% of all abortions in the year)	Misoprostol use (% of induced abortions in the year)
2006	67 (41.4)	1 (1.5)
2007	56 (39.2)	4 (7.1)
2008	71 (27.7)	5 (7.0)
2009	53 (24.2)	2 (3.8)
2010	64 (25.8)	4 (6.2)
2011	99 (25.3)	9 (9.1)
2012	106 (20.9)	13 (12.3)
2013	132 (38.2)	31 (23.5)
2014^a^	51 (28.1)	18 (35.3)
**Total**	**699**	**87**

^a^ Low figures due to national industrial actions for a significant part of the year

Cochran-Armitage test for linear trend: Chi-Square for Trend = 30.96, P <0.001

Compared to patients who used misoprostol, patients who procured abortion with other methods (and specifically, surgical abortion) had disproportionately more injuries to the genital tract, were more likely to have offensive vaginal discharge, pelvic or intra-abdominal abscesses and peritonitis and were more likely to present with high-grade fever and septicemia, or develop severe medical complications. Between these two groups of patients, there was no significant difference in the occurrence of retained products of conception, complications like severe anemia or hypotension, or mortality (Table 3).

**Table 3:** Comparison of clinical features and complications of abortions with misoprostol use to other methods, and specifically to surgical abortions.

Presentation	Abortion method		P value	Abortion method		P value
	Misoprostol (%)	Other methods(%)		Misoprostol (%)	Surgical abortion(%)	
High-grade fever	2 (3.6)	101 (16.3)	**0.010**^a^	2 (3.6)	70 (18.7)	**0.003**^a^
Offensive vaginal discharge	4 (7.0)	164 (25.9)	**0.001**^a^	4 (7.0)	106 (28.0)	**<0.001**^a^
Severe anaemia	9 (20.5)	81 (13.9)	0.229	9 (20.5)	49 (13.5)	0.213
Hypotension	5 (9.6)	88 (14.3)	0.350	5 (9.6)	55 (14.7)	0.323
Injuries to genital tract	0 (0)	89 (13.9)	**0.001**^a^	0 (0)	67 (17.3)	**<0.001**^a^
Retained products of conception	50 (87.7)	510 (91.9)	0.186	50 (87.7)	313 (82.4)	0.315
Intra-abdominal or pelvic abscesses	0 (0)	72 (11.2)	**0.002**^a^	0 (0)	53 (13.7)	**0.001**^a^
Peritonitis	0 (0)	90 (14.2)	**<0.001**^a^	0 (0)	67 (17.5)	**<0.001**^a^
Septicaemia	1 (1.8)	119 (18.7)	**<0.001**^a^	1 (1.8)	80 (20.9)	**<0.001**^a^
Medical complications ^b^	1 (1.8)	73 (11.4)	**0.022**^a^	1 (1.8)	45 (11.6)	**0.018**^a^
Mortality	0 (0)	8 (1.3)	>0.999^a^	0 (0)	5 (1.3)	>0.999^a^

^a^Fisher’s Exact test

^b^Includes renal failure, shock, disseminated intravascular coagulopathy, adult respiratory distress syndrome

Table 4 summarizes hospital interventions following the different methods used to procure an abortion. Compared to patients who used misoprostol to procure abortion, patients who had a surgical abortion were more likely to be admitted as in-patients, have prolonged course of antibiotics, were more likely to have a laparotomy, drainage of abscesses, and were more likely to require additional specialist care.

**Table 4:** Hospital interventions required following misoprostol use versus other methods, and specifically surgical abortions.

Intervention	Abortion method		P value	Abortion method		P value
	Misoprostol only (%)	Other methods (%)		Misoprostol only (%)	Surgical abortion only (%)	
In-patient care	36 (63.2)	592 (92.2)	**<0.001**	36 (63.2)	365 (94.3)	**<0.001**
Blood transfusion	16 (28.6)	235 (37.4)	0.191	16 (28.6)	148 (39.1)	0.131
Laparotomy	0 (0)	62 (9.7)	**0.006**^a^	0 (0)	46 (12.0)	**0.002**^a^
Drainage of intra-abdominal or pelvic abscesses	0 (0)	52 (8.1)	**0.016**^a^	0 (0)	39 (10.1)	**0.005**^a^
Repair of uterine perforation	0 (0)	27 (4.2)	0.157^a^	0 (0)	20 (5.2)	0.091^a^
Hysterectomy	0 (0)	9 (1.4)	>0.999^a^	0 (0)	5 (1.3)	>0.999^a^
Antibiotics (>7 days)	5 (8.8)	158 (24.8)	**0.006**	5 (8.8)	104 (27.0)	**0.003**
Admission to ICU/need for inotropic drugs	1 (1.8)	14 (2.2)	>0.999^a^	1 (1.8)	7 (1.8)	>0.999^a^
Renal dialysis	0 (0)	17 (2.7)	0.386^a^	0 (0)	10 (2.6)	0.623^a^
Additional specialist consultation	0 (0)	57 (9.0)	**0.010**^a^	0 (0)	38 (9.9)	**0.009**^a^

^a^Fisher’s Exact test

## Discussion

Almost three out of ten women managed for abortion at nine of the hospitals in south-west Nigeria had induced abortions. It is likely that this proportion is actually higher: some women are not likely to admit to interference, as abortions are restricted and they could be prosecuted, or at best, stigmatized [17]. Many of the local methods volunteered in this survey have long been used to induce abortions, with variable levels of success. Misoprostol is relatively new on the list, but as the trend shows, it is becoming increasingly popular.

It is not expected that presentations such as reproductive tract injuries would be associated with a medical abortion method such as misoprostol use, as found in this study. Other presentations like fever (and complications like infectious morbidity) were significantly less associated with misoprostol than with other methods and specifically, surgical abortions. It is reported that misoprostol abortions are not always complete [18–21]. This theoretically makes infectious morbidity a likely complication; however, the study did not corroborate this. Incomplete abortion was a common complication in this study, irrespective of the method used. These findings are in favor of use of misoprostol over surgical abortion, in agreement with other studies [21].

Studies comparing misoprostol to manual vacuum aspiration (MVA) for incomplete abortions have found that patients had less pain (though they bled somewhat more) and were more likely to choose misoprostol again in the same situation, and recommend it to a friend [19,20,22]. Completeness of abortion with misoprostol, however, is reported to be 92.2% to 98.8% compared to 100% with MVA [18–21]: this is imperfect, but acceptably high nonetheless. Singh [23] concluded from hospital studies that misoprostol reduces the severity in abortion complications. The finding of a relatively large percentage of women presenting with incomplete abortions to the selected hospitals in S.W. Nigeria may be a result of a number of factors. Firstly, the study is hospital-based so does not reflect the actual proportion of women who used misoprostol who subsequently presented for an incomplete abortion. Without a well-designed community-based study, it is not possible to estimate the use effectiveness of medical abortion in Nigeria, where abortion generally occurs outside a hospital setting. However, because women who wish to induce abortion, and who employ misoprostol, may be unsure of the outcomes of the use of the drug, this may result in them presenting at a hospital to ensure that the abortion is complete. This is also likely to occur because of the increased bleeding associated with misoprostol. Better methods of communicating the effects of the use of misoprostol use may result in decreases in the number of misoprostol users who subsequently seek surgical procedures to ensure that an abortion has been completed.

The WHO guidelines recommend that both surgical and medical abortions are safe and effective [24]. Abortions are safe “if they are done with a method recommended by WHO that is appropriate to the pregnancy duration and if the person providing or supporting the abortion is trained” [25]. However, as surgical abortions are usually procured illegally in Nigeria, they are often unsafe. An unsafe abortion occurs “when a pregnancy is terminated either by persons lacking the necessary skills or in an environment that does not conform to minimal medical standards, or both” [5]. The post-abortion care required for the studied subjects was significantly more elaborate when surgical abortions were procured, with associated morbidity and greater cost of care. It is also important to note that surgical complications are more common with dilatation and curettage (D & C), than the less traumatic MVA. D & C is more likely to be used in an illegal abortion. The study was unable to distinguish between the two methods, as patients were not able to report the types of instruments used for their procedures.

Most of the local studies in literature evaluated misoprostol’s effectiveness in treating incomplete abortions [19–21]. No studies assessing the effectiveness or safety of inducing abortions with misoprostol were found; Okonofua et al. [26] prospectively evaluated legally induced abortions with a combination of mifepristone and misoprostol. This makes the index study of some value, despite its limitations of being a retrospective, records-based study.

Other limitations of the study include missing data (as data sources were retrospectively-accessed medical records), and the possibility that some instances of misoprostol use may have been missed, as it depended on the woman’s ability to name or describe it. Also, the study assesses women who require health services following induced abortion. As previously discussed, these participants’ proportion among all women who procured abortions is not known, therefore, the comparisons made between misoprostol and surgical methods are limited. One cannot determine how many of either group did *not* need to seek post-abortion care.

It can be concluded that, in this study, severe complications, including infectious morbidity, are more likely to occur with surgical abortions than with misoprostol, and that misoprostol use has increased in recent years. It may be cautiously implied that misoprostol may be a safer method for abortion induction than traditional methods of abortion and surgical evacuation obtained in an illegal context. Its use for legally indicated abortion should be encouraged.

## Supporting information

S1 Appendix Data collection instrument. (DOCX)
