# Supplementary material for: Trends in misoprostol use and abortion complications: A cross-sectional study from nine referral hospitals in Nigeria
Source: PLoS One. 2018 Dec 31;13(12):e0209415. doi: 10.1371/journal.pone.0209415 (PMC6312220; doi:10.1371/journal.pone.0209415)
Supplement: S1 Appendix — (DOCX) [file pone.0209415.s001.docx]

**DEPARTMENT OF OBSTETRICS & GYNAECOLOGY**

**UNIVERSITY COLLEGE HOSPITAL, IBADAN**

**The Relationship Between the use of Misoprostol and the Type and Severity of Abortion Symptoms: A Multi-country Study**

**Standardized form for the collection of retrospective data**

| **Clinical Data Form** | | | | | | | | |
| --- | --- | --- | --- | --- | --- | --- | --- | --- |
| Identification number _______ | | |  | | | Hospital record no____________ | | |
|  |  | |  | | |  | |  |
| Outpatient consultation/Admission diagnosis ______________________________ | | | | | | | | |
|  | | | | | | | | |
| Condition on admission | | | Blood pressure ____________ | | | | | |
|  |  | | Temperature ______________ | | | | | |
|  |  | | Resuscitation required 1. Yes 2. No | | | | | |
|  |  | | Period of gestation _________ weeks | | | | | |
|  |  | |  | |  | | |  |
| In-patient | | | 1. Yes | | 2. No | | |  |
| Out-patient/Day care | | | 1. Yes | | 2. No | | |  |
|  |  | |  | |  | | |  |
| History of interference | | | None | | 1. Yes | | | 2. No |
|  |  | | Mifepristone | | 1. Yes | | | 2. No |
|  |  | | Misoprostol (oral/vaginal) | | 1. Yes | | | 2. No |
|  |  | | Other tablets | | 1. Yes | | | 2. No |
|  |  | | Mechanical interference | | 1. Yes | | | 2. No |
|  |  | | Other (specify) | | 1. Yes | | | 2. No |
|  |  | |  | |  | | |  |
| **Clinical symptoms and signs** | | | | |  | | |  |
| Temperature pattern | | |  | |  | | |  |
| At least two episodes of temperature of at least 38 degrees in 24 hours | | | | | 1. Yes | | | 2. No |
| Sustained temperature of at least 38 degrees over 24 hours | | | | | 1. Yes | | | 2. No |
| First assessment of Haemoglobin | | | | | _________ | | | percent |
| Offensive discharge per vaginum | | | | | 1. Yes | | | 2. No |
| Injuries to the vagina | | | | | 1. Yes (specify) | | | 2. No |
|  | | | | | __________________________ | | | |
| Injuries to the cervix | | | | | 1. Yes | | | 2. No |
| Foreign bodies in the vagina | | | | | 1. Yes (specify) | | | 2. No |
|  | | | | | __________________________ | | | |
| Cervical Os open | | | | | 1. Open | | | 2. Closed |
| Uterine size | | | | | 1. Yes | | | 2. No |
| Uterine tenderness | | | | | 1. Yes | | | 2. No |
| Retained products of conception | | | | | 1. Yes | | | 2. No |
| Pelvic abscess | | | | | 1. Yes | | | 2. No |
| Intra-abdominal abscess | | | | | 1. Yes | | | 2. No |
| Peritonitis | | | | | 1. Yes | | | 2. No |
| Uterine perforation | | | | | 1. Yes | | | 2. No |
| Septicemia | | | | | 1. Yes | | | 2. No |
| Septicemic shock | | | | | 1. Yes | | | 2. No |
| Hypovolenic shock | | | | | 1. Yes | | | 2. No |
| Renal failure | | | | | 1. Yes | | | 2. No |
| Disseminated Intravascular Coagulation (DIC) | | | | | 1. Yes | | | 2. No |
| Adult Respiratory Distress Syndrome (RDS) | | | | | 1. Yes | | | 2. No |
|  |  | |  | |  | | |  |
| **Management** |  | |  | |  | | |  |
| Infusion | | |  | | 1. Yes | | | 2. No |
|  |  | | If yes | | Two units of 500 ml | | | _________ |
|  |  | |  | | More than two units of 500 ml | | | _________ |
| Blood transfusion | | |  | | 1. Yes | | | 2. No |
|  |  | | If yes | | Two or less units | | | _________ |
|  |  | |  | | More than two units | | | _________ |
|  |  | |  | |  | | |  |
| **Operative Procedures** | | |  | |  | | |  |
| Misoprostol for cervical ripening | | | | | 1. Yes | | | 2. No |
| E and C | | | | | 1. Yes | | | 2. No |
| MVA | | | | | 1. Yes | | | 2. No |
| D and C | | | | | 1. Yes | | | 2. No |
| Colpotomy | | | | | 1. Yes | | | 2. No |
| Laparotomy | | | | | 1. Yes | | | 2. No |
| Drainage of pelvic abscess | | | | | 1. Yes | | | 2. No |
| Drainage of abdominal abscess | | | | | 1. Yes | | | 2. No |
| Hysterectomy | | | | | 1. Yes | | | 2. No |
| Repair of uterine perforation | | | | | 1. Yes | | | 2. No |
| Other procedures | | | | | 1. Yes (specify) | | | 2. No |
|  |  | |  | | ___________________________ | | | |
|  |  | |  | |  | | |  |
| **Antibiotics** | | |  | |  | | |  |
| Oral for 7 days | | | | | 1. Yes | | | 2. No |
| Parenteral plus oral for up to 7 days | | | | | 1. Yes | | | 2. No |
| Parenteral plus oral for more than 7 days | | | | | 1. Yes | | | 2. No |
|  | | |  | |  | | |  |
| **Other medical procedures** | | | | |  | | |  |
| Admission to Intensive Care Unit (ICU) | | | | | 1. Yes | | | 2. No |
| Renal dialysis (refer to renal unit/hospital) | | | | | 1. Yes | | | 2. No |
| Inotropic drugs given | | | | | 1. Yes | | | 2. No |
|  |  | |  | |  | | |  |
| Additional specialist consultation | | | | | 1. Yes (specify) | | | 2. No |
|  |  | |  | | ___________________________ | | | |
| Referral to another department/hospital | | | | | _____________ | | | Name |
| Mortality | 1. Yes | | 2. No | | If yes, state cause _____________ | | | |
| Discharge diagnosis | | | _________________________________________ | | | | | |
| Discharge status | | 1. Discharged with permission of Doctor | | 2. Discharged on own request | | | 3. Discharged on own request (in moribund condition) | |
